# Long term efficacy and safety of Fludarabine, Cyclophosphamide and Rituximab regimen followed by ^90^Y-ibritumomab tiuxetan consolidation for the treatment of relapsed grades 1 and 2 follicular lymphoma

**DOI:** 10.1186/s40164-015-0012-3

**Published:** 2015-06-24

**Authors:** Francesco Pisani, Rosa Sciuto, Maria Laura Dessanti, Diana Giannarelli, Ramy Kayal, Sandra Rea, Francesco Marchesi, Mirella Marino

**Affiliations:** Department of Hematology Regina Elena National Cancer Institute, Via Elio Chianesi, 53 - 00144 Rome, Italy; Department of Nuclear Medicine Regina Elena National Cancer Institute, Rome, Italy; Unit of Hematology and Stem Cell Transplant Azienda Ospedaliera San Camillo-Forlanini, Rome, Italy; Biostatistics Regina Elena National Cancer Institute, Rome, Italy; Department of Radiology Regina Elena National Cancer Institute, Rome, Italy; Department of Pathology Regina Elena National Cancer Institute, Rome, Italy

**Keywords:** Follicular lymphoma, ^90^Y-ibritumomab tiuxetan, Radioimmunotherapy

## Abstract

**Background:**

In this retrospective study, we investigated the efficacy and safety of radioimmunotherapy with ^90^Yttrium- ibritumomab tiuxetan (^90^Y-RIT) in 9 patients with recurrent follicular lymphoma (FL) who were treated in a consolidation setting after having achieved complete (CR) or partial remission (PR) with Fludarabine, Cyclophosphamide and Rituximab (FCR).

**Methods:**

The median age was 63 years (range 46–77). All patients were relapsed with histologically confirmed CD20-positive (grade 1 or 2) FL, at relapse they received FCR every 28 days: F (25 mg/m^2^x 3 days), C (1 gr/m^2^ day 1) and R (375 mg/m^2^ day 4) for 4 cycles. Those who achieved at least a PR with <25 % bone marrow involvement were treated with ^90^Y-RIT 11.1 or 14.8 MBq/Kg, at 3 months after completing FCR. Patients underwent a further restaging at 12 weeks after ^90^Y-RIT with a total body CT scan, FDG-PET/CT and bilateral bone marrow biopsy.

**Results:**

Nine patients completed the treatment: FCR followed by ^90^Y-RIT (6 patients at 14.8 MBq/Kg, 3 patients at 11.1 MBq/Kg). After FCR, 7 patients obtained CR and 2 PR; after ^90^Y-RIT 2 patients in PR converted to CR 12 weeks later. With a median follow up of 95 months (range 20–114) since FCR and 88 months (range 13–104) since ^90^Y-RIT 3 deaths were not related to lymphoma; all 3 deceased patients obtained CR before ^90^Y-RIT and died still in CR. The median overall (OS) and progression free survival (PFS) have not been reached, in this analysis both OS or PFS are 67 % at 7.5 year. The most common grade 3 or 4 adverse events were hematologic.

**Conclusions:**

These results confirm the long term efficacy and safety of 4 cycles of FCR followed by ^90^Y-RIT in relapsed grades 1 and 2 FL and suggest that this regimen could be a therapeutic option for this setting of patients, specially at age of 60–75 with no unexpected toxicities.

## Introduction

Most cases of follicular lymphoma are characterized by recurrence of disease. There is usually a pattern of repeated remissions and relapses until patients become refractory to treatment. The duration of remissions becomes shorter with repeated induction attempts. Transformation to more aggressive non-Hodgkin lymphoma (NHL) occurs in 15 % to 50 % of the patients at 5 years. Therefore, it is important to have many treatment options: combination chemotherapy, radiation, immunotherapy, radioimmunotherapy and myeloablative therapy with stem-cell rescue for some patients with good performance status and responsive disease to overcome the development of resistance. A number of cytotoxic agents in combination are active in this patient population. The fludarabine, cyclophosphamide and rituximab (FCR) regimen provided encouraging results as initial or salvage therapy in patients with CLL or indolent NHL [1, 2]. Radioimmunotherapy is also an excellent modality in the treatment of NHL; the target antigen, radionuclide emission properties and chemical stability of radioimmunoconjugates are important factors that contribute to the effectiveness of RIT. ^90^Y can deliver a high beta energy to tumor (2–3 MeV) and ^90^Yttrium- ibritumomab tiuxetan (^90^Y-RIT ) consists of the anti-CD20 monoclonal antibody ibritumomab (an IgG1k antibody which is the murine parent immunoglobulin to rituximab) covalently bound to the chelating agent tiuxetan and radiolabeled with ^90^Y.

The phase III FIT trial (First-line Indolent Trial), enrolled 414 patients with stage III or IV who had attained a CR or PR after induction chemotherapy. It showed that consolidation of first remission with ^90^Y-RIT was highly effective with no unexpected toxicities, producing a statistically significant longer time to progression in both PR and CR patients groups. In the last update of the trial the median PFS has not yet been reached (>7.9 years) for patients in the ^90^Y-RIT arm and 4.9 years in control arm [3–5]. Furthermore, several phase II trials show high rates of conversion from PR to CR and significant improvements in PFS [6–14] using consolidation therapy with ^90^Y-RIT obtained, after initial treatment. ^90^Y-RIT also has been reported to be effective in patients with relapsed or refractory FL [15–17]. Here, we report updated long–term efficacy and toxicity results of ^90^Y-RIT consolidation in 9 patients relapsed with grade 1 and 2 FL patients responding to FCR that were treated at our Institute [18].

## Results

### Patients characteristics

In this retrospective analysis 9 patients had received 4 cycles of FCR followed by ^90^Y-RIT (6 patients at 14.8 MBq/Kg, 3 patients at 11.1 MBq/Kg). Baseline characteristics are presented in Table 1. The median age was 63 years (range 46–77), all patients were relapsed patients: 2 patients received a prior therapy, 5 patients received 2 prior treatments and 2 patients received 3 regimens. Seven patients were previously treated with rituximab plus chemotherapy, 2 patients had no previous rituximab treatment history, 1 patient received also high-dose therapy followed by autologous stem cell transplantation (Table 2).

**Table 1:** Patient characteristics

		Patients (*n* = 9)
Male/Female		3/6
Median Age (Range)		63 (46–77) years
Disease stage	at diagnosis	at start of FCR
I	1	0
II	1	5
III	1	3
IV	6	1
Bone marrow involvement		
0 %		7
10 % to ≤25 %		2
Extranodal involvement		1 (liver)
FLIPI		
Low		1
Low-intermediate		6
Intermediate-high		2
Bulky disease		1
B-symptoms		0
Previous therapy including rituximab		
No		2
Yes		7
Number of previous regimens		
1		2
2		5
> 2		2

**Table 2:** Clinical characteristics

Patients *n*	Sex/Age (y)	Previous treatment	Response to FCR	Response to RIT	Follow up (mo) since RIT
1	F/68	CHOP/R,radiotherapy	CR	CR	104 alive in CR
2	F/66	Radiotherapy, CHOP/R	CR	CR	88 alive in CR
3	F/57	CHOP/R	PR	CR	99 alive in CR
4	F/67	CHOP/R, radiotherapy	CR	CR	13 dead in CR
5	M/46	CHOP/like, ASCT, IFN maintenance for 24 months	PR	CR	83 alive in CR
6	F/61	MACOPB/R	CR	CR	92 alive in CR
7	M/69	CHOP, FM/R, CyDex/R	CR	CR	30 dead in CR (t-MDS)
8	M/57	Chlorambucil, MACOPB/R	CR	CR	32 dead in CR
9	F/77	Chlorambucil, radiotherapy	CR	CR	99 alive in CR

*CHOP* cyclophosfamide, doxorubicin, vincristine, prednisone; *R* Rituximab; *MACOPB* Methotrexate, Doxorubicin, cyclophoshamide, vincristine, prednisone, bleomycin; *ASCT* autologous stem cell transplantation; *IFN* alpha interferon, *FM* fludarabine, mitoxantrone; *Cy Dex* cyclophosphamide, dexamethasone; *t-MDS* treatment-related myelodysplastic syndrome

### Efficacy and safety

**Fig. 1:**
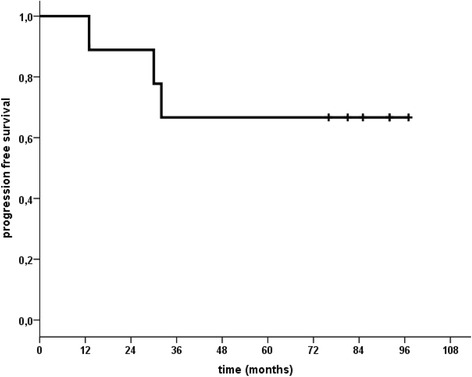
Progression Free Survival from RIT

After 4 cycles of FCR 7 patients obtained CR and 2 PR, 2 patients in PR converted to CR after ^90^Y-RIT. In February 2015, with a median observation period of 95 months (range 20–114) since FCR and 88 months (range 13–104) since RIT, the median OS and the PFS have not been reached, 6/9 patients were alive in CR and current analysis has shown that either OS or PFS are 67 % at 7.5 year (Fig. 1). Grade 3 or 4 neutropenia occurred in 8/9 patients treated with FCR and in 9/9 patients assessable after ^90^Y-RIT. Subsequently to radioimmunotherapy the median neutrophil nadir was 0.8 × 10^9^/ L (range 0.1-0.9 × 10^9^/ L) at week 5, the median platelet count nadir was 49 × 10^9^/ L (range 17–80 × 10^9^/ L) at week 5. The median duration nadir for both neutrophils or platelets was 14 days. One patient developed herpes zoster infection after 8 months following valacyclovir discontinuation; another patient developed fungal infection. Both infections disappeared after specific treatment. One patient developed t-MDS (treatment-related myelodysplastic syndrome) at 26 months after ^90^Y-RIT. This patient before FCR and consolidation with ^90^Y-RIT had received 3 previous regimens: at diagnosis 6 courses of CHOP, at first relapse, 3 years later, 4 courses of FM/R (fludarabine, mitoxantrone plus rituximab) and after 1 year at the second relapse the patient received cyclophosphamide plus dexamethasone and rituximab, remaining in CR for 48 months. The patient died at 73 years of age of sepsis during support therapy for t-MDS. Other 2 patients died: 1 for acute renal failure and 1 for ictus cerebri.

## Discussion

Cytotoxic chemotherapies lose efficacy with subsequent rounds of therapy in the retreatment of follicular lymphoma, eventually leading to refractory disease, however the question remains whether the survival of patients with FL is improving with new treatment regimens.

In the current retrospective analysis, nine patients with relapsed grade 1 and 2 FL, responding to FCR regimen and consolidated with ^90^Y–RIT obtained a significant high rate of response with 100 % of CR and acceptable toxicity. The conversion from PR to CR was already shown in the published phase III study (FIT-study) in first-line FL [3, 4] and also in phase II studies [5–12] of consolidation with the radioimmunotherapy agent ^131^ I-tositumomab after first-line induction [19, 20] thus, confirming the ability of ^90^Y-RIT to improve responses also in patients who are pretreated with rituximab based combination therapy [3]; even if in our two patients there is no proof that this conversion was due to RIT and not to a late response to FCR. In the FIT study, close to 17 % of the patients in the control arm, converted from PR to CR during watchful waiting [3], but our 2 patients who had higher risk of resistance already being pretreated must be considered.

In our analysis, the OS at 2 years was 89 %, at 3 years 76 % and at 4 years 61 % and OS and PFS are 67 % at 7.5 years. In another study conducted on patients with recurrent FL, treated with FCR, 75 % OS rate at 4 years and 61 % PFS rate at 4 years were registered, but in that study only 7 % of patients had been treated previously with rituximab and furthermore no patients had received combination treatment with chemotherapy plus rituximab [21]. Furthermore our results are in line with those recently described in a Japanese study [22] on 94 patients with relapsed or refractory low grade B cell non-Hodgkin lymphoma, among them 61 patients with grade 1 and 2 FL, treated with ^90^Y-RIT alone as salvage therapy and showing a CR rate of 69 %. In the Japanese cohort, during a median follow up of 46.5 months, the PFS rates of the first 50 patients who had undergone ≤ 2 and ≥ 3 previous regimens, and for those who achieved CR compared with those who did not were 38 and 11 months, respectively; the number of previous regimens and CR were statistically significant (*p* = 0.0011 and *p* < 0.0001, respectively). In our study 7/9 patients underwent ≤ 2 previous regimens before FCR and all of patients reached CR after ^90^Y-RIT with a PFS of 67 % at 7.5 years. Regarding AEs no grade 3 or 4 anemia was noted and no erythropoietic growth factors were used; there was high incidence of grade 3 or 4 neutropenia and thrombocytopenia but no platelet transfusions were necessary and granulocyte colony-stimulating factors were utilized in the majority of patients during FCR treatment and in all of them after ^90^Y–RIT. Despite the high incidence of grade 3 or 4 neutropenia, there were no patients requiring hospitalization for infection. We registered a case of herpes zoster infection after 8 months following valacyclovir discontinuation that disappeared after retreatment, and a case of fungal infection by *conidiobolus,* developed 10 months after ^90^Y-RIT and disappeared with itraconazole treatment. Other previous studies have already shown the low percentage of patients requiring hospitalization for infections [3, 15] and a favorable safety profile [23, 24]. A case of t-MDS with complex karyotype was diagnosed 26 months after ^90^Y-RIT consolidation: this patient received 3 previous regimens before FCR plus ^90^Y-RIT and as already mentioned the patient died of sepsis. This patient had been previously treated with topoisomerase II inhibitors, alkylating agents and purine nucleoside analogs. Czuczman et al. reported incidence of t-MDS and t-AML (treatment-related acute myeloid leukemia) after ^90^Y–RIT of 0.3 % per year after the diagnosis of NHL and 0.7 % per year after treatment. Most patients with t-MDS or t-AML had multiple cytogenetic aberrations, commonly on chromosomes 5 and 7, suggesting an association with previous exposure to chemotherapy. In Czuczman study, these malignancies were diagnosed at a median of 5.6 years (range 1.4 to 13.9) after the diagnosis of NHL and 1.9 years (range 0.4 to 6.3) after radioimmunotherapy [25]. The conclusion of this study was that the annualized incidences of t-MDS and t-AML were consistent with that expected in patients with NHL who had extensive previous chemotherapy and did not seem to increase after ^90^Y-RIT. However, in the FIT study 8 patients who developed MDS/AML were treated with ^90^Y-RIT, suggesting a role played by ^90^Y-RIT in the risk of secondary MDS/AML, thus it is reasonable to consider monitoring these patients closely. Cytogenetic testing before treatment with RIT may identify existing chromosomal abnormalities in previously treated patients, particularly those who have been treated with alkylating agents and purine analogs and would be at higher risk of developing t-MDS or t-AML.

In our series, the other two deaths were not related to progressive disease and all three deceased patients obtained CR before ^90^Y-RIT and died still in CR; so far the six survivors have maintained a high quality of life without having to make many visits to the hospital due to toxicity. Additional follow up is required to determine potential long-term AEs with ^90^Y-RIT consolidation. In our patients, the response to ^90^Y-RIT was assessed by CT, bone marrow biopsies and also with FDG-PET. This imaging procedure is useful to evaluate disease extension before treatment and response to RIT in FL. A study has shown that the post-^90^Y–RIT PET result is an independent predictive factor of PFS [26].

## Conclusions

This retrospective analysis of 9 relapsed grades 1 or 2 FL heavily pretreated patients with median age 63 years demonstrates that sequential treatment with FCR and ^90^Y-RIT did not give rise to cumulative toxicity; it was feasible, safe and yielded high OS and PFS in patients with recurrent FL. Hematologic toxicity occurring with FCR or with ^90^Y-RIT was clinically controllable and acceptable in a population composed mainly of patients with a history of prior treatment using rituximab plus chemotherapy. With caution due to the low number of patients, these results suggest that this regimen could be an option used for the treatment in this setting of patients, specially at age of 60–75. ^90^Y-RIT as consolidation appears to be best suited to patients with low burden of disease and may be more acceptable in those who are not candidates for high dose therapy/transplant approaches.

### Design and methods

**Fig. 2:**
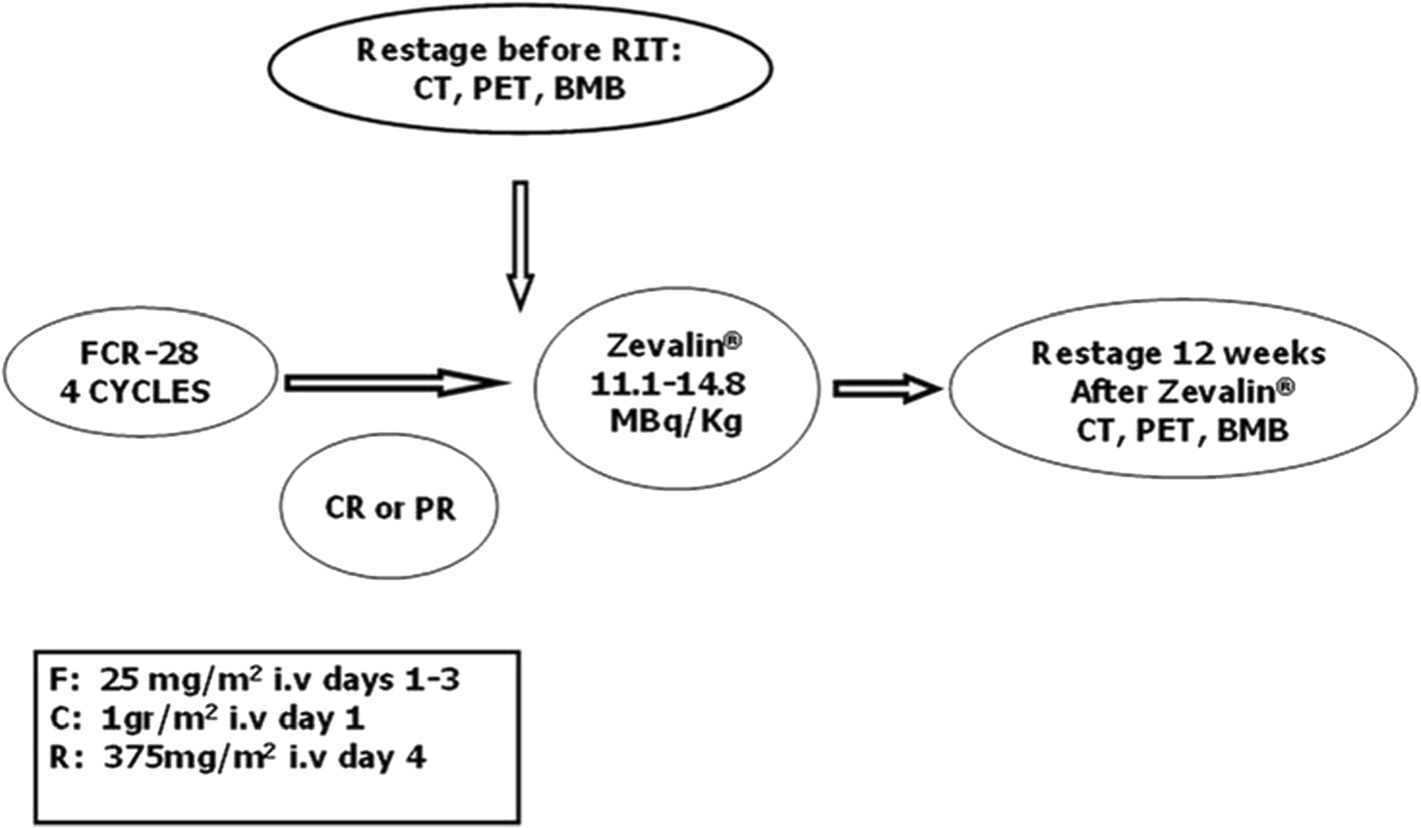
Treatment schema

The patients who were included in the current retrospective analysis had CD20+ histologically confirmed relapsed grade 1 or 2 follicular lymphoma and had received at least 1 prior treatment. In this single institution study, between August 2005 and July 2010, 9 patients at relapse had received 4 cycles of FCR: fludarabine at a dose of 25 mg/m^2^ i.v. on days 1 to 3; cyclophosphamide at a dose of 1 gr/ m^2^ i.v. on day 1 and rituximab at a dose of 375 mg/ m^2^ was given on day 4 of each cycle every 28 days. Patients were restaged with CT scan, FDG PET/CT and bone marrow biopsies after the last course of FCR, who had achieved at least a partial remission with < 25 % bone marrow involvement received, 12 weeks since the last course of FCR, 2 infusions of rituximab 250 mg/ m^2^ one week apart, with the first infusion administered alone and the second infusion followed immediately by ^90^ Y–RIT (14.8 MBq/Kg – 11 MBq/Kg), if the platelet number was between 100 x 10^9^/ L and 149 x 10^9^/ L, not exceeding a total of 1.184 MBq it was administered as a slow i.v. push over 10 min (Fig. 2). The patients were age ≥ 18 years, with WHO performance status of 0 to 2 and the last chemotherapy with or without rituximab was administered at least 3 months before start of FCR; no patient under maintenance therapy with rituximab was considered. Before starting ^90^Y-RIT an absolute neutrophil count ≥ 1.5 × 10^9^ L, hemoglobin levels ≥ 9 gr/dl and a platelet count ≥ 100 × 10^9^ L were required. None of the patients had central nervous system (CNS) involvement and positive HIV. All patients provided an informed consent according to institutional guidelines.

No real-time quantitative PCR (RQ-PCR) evaluation of peripheral or marrow blood samples for bcl-2 t(14;18) translocation was performed at baseline nor thereafter. Safety was assessed by adverse events (AEs), with toxicity grading based on the National Cancer Institute Common Toxicity Criteria (version 4.0), clinical laboratory evaluations, and physical examinations. Filgrastim was administered when the neutrophil count was less than 1×10^9^/L and platelet support was planned for eventual episodes of bleeding and platelet count less than 15×10^9^/L. In patients developing grade 4 neutropenia or thrombocytopenia, the duration of cytopenia was measured from the first day of laboratory evidence of grade 4 toxicity until the last day of grade 4 toxicity without further support. OS was calculated from the date of FCR treatment to the date of death from any cause; OS was analyzed by using the Kaplan-Meier method.

## Abbreviations

FCR: Fludarabine cyclophosphamide rituximab
FL: Follicular lymphoma
NHL: Non-Hodgkin lymphoma
RIT: Radioimmunotherapy
MeV: Megaelectronvolt
MBq: Megabecquerel
OS: Overall survival
PFS: Progression free survival
t-MDS: Treatment related myelodysplastic syndrome
